# Diflubenzuron Did Not Affect the Abilities of the Backswimmer *Buenoa tarsalis* to Survive and Prey Upon Larvae of *Aedes aegypti*

**DOI:** 10.3390/insects16040435

**Published:** 2025-04-21

**Authors:** Sabrina H. C. Araujo, Luis G. Salinas Jimenez, Maria J. M. Corrêa, Viviana L. Bohorquez Zapata, Monalisa S. S. Oliveira, Joshua S. Fernandes, Jônatas M. Gomes, Raimundo W. S. Aguiar, Gil R. Santos, Wilson R. Valbon, Eugênio E. Oliveira

**Affiliations:** 1 Departamento de Entomologia, Universidade Federal de Viçosa, Viçosa 36570-900, MG, Brazil; 2 University of California Malaria Initiative (UCMI), National Center of Endemic Diseases, Sao Tome, Sao Tome and Principe; 3 Tecnoacademia Manizales, Servicio Nacional de Aprendizaje (SENA), Regional Caldas, Manizales 170003, Caldas, Colombia; 4 Departamento de Farmácia, Universidade Federal do Ceará, Fortaleza 60430-370, CE, Brazil; 5 Programa de Pós-Graduação em Biotecnologia, Universidade Federal do Tocantins, Gurupi 77410-530, TO, Brazil; 6 Programa de Pós-Graduação em Ciências Florestais, Universidade Federal do Tocantins, Gurupi 77402-970, TO, Brazil

**Keywords:** *Belostoma anurum*, *Poecilia reticulata*, non-target organisms, unintended effects, insect growth regulators

## Abstract

Diflubenzuron is a growth regulator insecticide and acaricide commonly used to control the immature stages of agricultural pests and mosquitoes that vector relevant animal diseases. Despite the beneficial effects of diflubenzuron, residues of these molecules can reach the aquatic ecosystems, which may lead to detrimental effects on aquatic non-target organisms. Here, we evaluated whether exposures to diflubenzuron (0.267 mg active ingredient [a.i.]/L, which is the equivalent to field dose recommendation) would affect the survival abilities of three non-target aquatic predators: adults of the backswimmer *Buenoa tarsalis* (Hemiptera: Notonectidae), nymphs of the water bug *Belostoma anurum* (Hemiptera: Belostomatidae), and adults of the guppy fish *Poecilia reticulata* (Cyprinodontiformes: Poeciliidae). We further evaluated potential undesired sublethal effects by assessing the abilities of *Bu. tarsalis* to prey upon second instar larvae (L2) of *Aedes aegypti* (Diptera: Culicidae) when exposed to the no-observed-lethal concentration (NOLC, 267 mg active ingredient [a.i.]/L., which corresponded to 1000-fold the field dose recommendation). Backswimmer adults were exposed to two sublethal exposure (24 h and 96 h) conditions and three prey densities (i.e., three, six, and nine larvae/100 mL) using artificial water reservoirs. Our results revealed that diflubenzuron (0.267 mg [a.i.]/L) was not lethal to any non-target aquatic predators. Furthermore, exposure (24 h and 96 h) to diflubenzuron at concentrations of 267 mg [a.i.]/L was unable to disturb the ability of *Bu. tarsalis* to survive and to prey upon *Ae. aegypti* larvae at all prey densities. Altogether, our findings demonstrate that diflubenzuron exhibits negligible ecological risks to naturally occurring mosquito predators and represents a safe tool to be utilized in aquatic ecosystems.

## Simple Summary

Diflubenzuron is a widely used insecticide. It is used on a variety of target organisms, including mosquitoes’ larvae. However, the misuse of diflubenzuron near and in aquatic environments can lead to unintended effects to beneficial organisms that inhabit these places. Here, we evaluated the impacts of diflubenzuron on two species of water bugs, *Buenoa tarsalis* and *Belostoma anurum*, and on the guppy fish *Poecilia reticulata*. Exposure to the insecticide at concentrations equivalent to the recommended field dose had no impact on the survival of the species. Even at 1000-fold higher concentrations, diflubenzuron was unable to impact on the ability of *Bu. tarsalis* to prey upon larvae of the mosquitoes *Aedes aegypti*. The results reinforce the safety of diflubenzuron for non-target aquatic organisms capable of preying on mosquitoes’ larvae.

## 1. Introduction

The management of mosquitoes that transmit human diseases such as Zika, dengue fever, and chikungunya relies mainly on the abatement of immature stages by larvicidal insecticides [1,2]. The benzoylphenyl urea diflubenzuron is a commonly used insecticide (and acaricide) belonging to the class of insect growth regulators (IGRs) that target arthropod immature stages. It is a chitin synthesis inhibitor that acts by blocking the synthesis of chitin, which is a key component of the exoskeleton in arthropods [3]. More specifically, diflubenzuron interacts with chitin synthesis 1 (CHS1), leading to an early molting process without a properly formed exoskeleton, which results in the death of immature stages of the target organisms, such as mosquito larvae [4,5]. Although it has been effective against a diverse range of insects and mites, previous studies have demonstrated that diflubenzuron has low to moderate toxicity to non-target organisms, including natural predators of mosquito larvae [6,7,8]. Such ecotoxicological studies, however, are limited to lethal effects of diflubenzuron, and little attention is paid to potential sublethal effects on the physiology or behavior of aquatic predators.

Besides being used to control mosquitoes in open larval habitats [2,8], diflubenzuron is also applied to control fish parasites in aquaculture [9]. However, in some cases, the implementation of diflubenzuron by fish farmers does not follow the appropriate recommendations and possibly results in indiscriminate and non-standardized applications with highly variable concentrations [10]. Thus, the incorrect use of diflubenzuron may be detrimental to beneficial aquatic organisms, such as detritivore and predator insects, that inhabit aquaculture ecosystems.

Among the beneficial organisms in freshwater reservoirs, the mosquito’s predators contribute to shaping community structure, not only by preying upon mosquito larva but also by serving as prey of vertebrates and other invertebrates [11]. The complexities of these trophic interactions in aquatic ecosystems might be unbalanced by the presence of abiotic stressors, such as synthetic and biorational insecticides [12,13,14,15]. Among the aquatic mosquito predators, the semiaquatic backswimmer (Hemiptera: Notonectidae) represents an interesting model organism, as these insects voraciously prey upon mosquito larvae and are relatively tolerant to non-neurotoxic larvicides commonly used in mosquito control [6,8,16,17]. For instance, pyriproxyfen, an insect growth regulator insecticide, not only showed a safe margin of selectivity to the backswimmer *Buenoa amnigenus* (White) (Hemiptera: Notonectidae), but exposure to sublethal levels also did not alter the ability of *Bu. amnigenus* to prey upon mosquito larvae [17]. Similarly, the water bugs (Hemiptera: Belostomatidae) are cosmopolitan and long-lived organisms that prey upon mosquito larvae in natural and artificial water reservoirs [11,18,19,20]. Recent studies, however, have shown that not only are belostomatids susceptible to mosquito larvicides, but, also, their physiology and behavior are affected by sublethal exposure to insecticides [21,22,23]. Vertebrate predators, such as larvivorous fish, also play an important role in the biological control of mosquito species. Interestingly, larvivorous fish have been adopted worldwide for mosquito control in aquatic habitats [24,25,26]. However, these predators also show some levels of susceptibility to mosquito larvicides such as diflubenzuron [7,27]; thus, an appropriate selection of insecticide is required if used in combination with larvivorous fish in mosquito control programs.

Given the adverse effects of larvicidal insecticide on non-target organisms, we hypothesized that diflubenzuron is toxic to naturally occurring mosquito predators in freshwater ecosystems. Further, sublethal diflubenzuron exposure impairs the ability of backswimmers to prey upon mosquito larvae in artificial containers. Specifically, we evaluated (1) whether diflubenzuron (at a concentration equivalent to the field recommendation for mosquito control) is lethal to adults of backswimmer *Buenoa tarsalis*, nymphs of water bug *Belostoma anurum*, and adults of guppy fish *Poecilia reticulata* (Cyprinodontiformes: Poeciliidae); (2) whether *Bu. tarsalis* adults exposed sublethal levels of diflubenzuron show impaired abilities to prey upon *Aedes aegypti* larvae at different prey densities.

We used the yellow fever mosquito *Ae. aegypti* as the model prey, based on findings described elsewhere [11,18,20,26], which demonstrated that *Ae. aegypti* larvae occur in both natural and artificial water reservoirs and have the potential to be preyed upon by generalist predators.

## 2. Materials and Methods

### 2.1. Fish and Insects

Adults of the backswimmer *Bu. tarsalis* were collected in fish farming facilities (Viçosa, MG, Brazil, 20°45′ S, 42°52′ W). Previous to the bioassays, backswimmers were acclimated for 24 h in a 600 mL glass beaker containing 500 mL of distilled water (conductivity of 236 ± 9.5 μS/cm and pH of 7.1 ± 0.2) under controlled conditions (25 ± 2 °C, 12 h of photophase). The backswimmer specimens were anaesthetized with ice (5 min) and identified using a stereomicroscope, following previously described taxonomic keys [28]. The body length of *Bu. tarsalis* adults was 6.8 ± 3.8 mm (n = 10). We also used second instar nymphs of water bug *Be. anurum*, obtained from a laboratory population that was reared under controlled conditions for more than 8 years. Briefly, nymphs and adults of *Be. anurum* were reared in dechlorinated tap water under controlled temperature (25 ± 2 °C) and photoperiod (12:12 L:D) conditions. The first and second instar nymphs of *Be. anurum* were fed upon *Ae. aegypti* larvae, and adults were fed upon notonectids and guppy fish [20,29]. The body length of the second instar *Be. anurum* nymphs was 6.9 ± 0.2 mm (n = 10). Adults of guppy fish *Po. reticulata* were collected in the fish farming facilities (Viçosa, MG, Brazil, 20°45′ S, 42°52′ W) and acclimated for 96 h under controlled conditions (25 ± 2 °C and photoperiod of 12 h) before being used in the experiments. Adults of the guppy fish (n =10) exhibited a body length of 42.3 ± 10.3 mm. Second instar larvae (L2, body length of 2.9 ± 0.1 mm, n = 10) of a pyrethroid-susceptible *Ae. aegypti* strain (PP-Campos, Campos dos Goytacazes, RJ, Brazil) were obtained from a laboratory population maintained under controlled conditions [20,30]. For all cases, we reared the insects and fish in an environment free of insecticides.

### 2.2. Survival Bioassays

We assessed the susceptibility of three naturally occurring mosquito larvae predators, *Bu. tarsalis* adults, *Be. anurum* nymphs, and *Po. reticulata* adults, to diflubenzuron (Dimilin 80 WG, 800 g a.i./kg; Arysta Lifescience, São Paulo, Brazil). We used unsexed adults for *Bu. tarsalis* and *Po. Reticulata*. Regarding the insecticide concentrations, we started from the insecticide concentration of 0.267 mg a.i./L, which corresponds to the concentration recommended to control mosquito larvae, up to 267 mg a.i./L (i.e., 1000-fold the recommendation for field applications). For all survival bioassays, control treatments were conducted, exposing the insects (*Be. anurum* and *Bu. tarsalis*) and fish (*Po. reticulata*) to an insecticide-free distilled water (conductivity of 236 ± 9.5 μS/cm and pH of 7.1 ± 0.2) solution. The control group of the selected organisms was maintained in the experimental medium/environment and controlled conditions for the duration of the experiment.

For *Bu. tarsalis* adults, our experimental unit consisted of a group of 10 insects placed into five 250 mL glass vials (7.0 cm diameter × 9.0 cm height) containing 100 mL of insecticide solutions, either at 0.267 mg a.i./L or 267 mg a.i./L, which correspond to 1- and 1000-fold the concentration recommended for field applications for controlling mosquito (*Aedes* sp. and *Anopheles* sp.) larvae [1,2]. We used five repetitions for each insecticide concentration, which totalized 50 *Bu. tarsalis*. Mortality was assessed every 24 h for 13 consecutive days, and insects that remained motionless after being repeatedly stimulated mechanically with a pipette were considered dead [17]. For the survival assay with *Be. anurum* nymphs, we placed a sole individual per glass vial, in order to avoid cannibalism. Each 250 mL glass vial (7.0 cm diameter × 9.0 cm height) contained 100 mL of solution at 0.267 mg a.i/L. A total of 30 nymphs (i.e., three groups of 10 individualized nymphs as replicates) was used. The *Be. anurum* nymphs’ mortalities were assessed every 24 h for 13 consecutive days. All glass vials were covered with organza fabric to avoid insect escape [21].

For *Po. reticulata* bioassays, we used 10 L glass (20.5 cm height × 28.0 cm diameter) aquaria containing 500 mL of 0.267 mg a.i/L diflubenzuron solution. We used three aquaria containing 20 individuals each, totalizing 60 fish per treatment. The aquaria were maintained under constant aeration. Fish mortality was assessed daily for four consecutive days and dead individuals were removed from the container [31]. All survival bioassays were performed at controlled temperature (25 ± 2 °C), humidity (60 ± 2%), and photoperiod (12 h of light phase) conditions.

### 2.3. Predation Bioassays with Bu. tarsalis Adults

To assess the impact of diflubenzuron sublethal levels on the abilities of *Bu. tarsalis* adults to prey upon *Ae. aegypti* larvae (L2), we conducted two experimental sets. First, we tested short-term sublethal exposure, where insects were exposed to diflubenzuron at a concentration of 267 mg a.i./L for 24 h. Later, a new group of *Bu. tarsalis* were diflubenzuron (267 mg a.i./L)-exposed for 96 h, which represents a longer-term exposure scenario. We selected 267 mg a.i./L as a sublethal level based on our survival experiment, which did not show any effect on *Bu. tarsalis* survivorship compared to unexposed insects. Similar to the survival bioassay described above, *Bu. tarsalis* adults were exposed to diflubenzuron using 100 mL of the solution in glass vials (7.0 cm diameter × 9.0 cm height) of 250 mL volumetric capacity. The control treatments were conducted by exposing the insects to distilled water only for 24 h and 96 h, and, later, they were transferred to new glass vials containing 100 mL distilled water to start the predation experiment. After each diflubenzuron exposure (i.e., 24 or 96 h), insects were individually transferred into glass containers (7.0 cm diameter × 9.0 cm height) containing 100 mL of distilled water and left for 1 h to acclimatize. Later, L2 *Ae. aegypti* larvae were offered to each individualized *Bu. tarsalis* in one of three larval densities (three, six, or nine larvae/100 mL of distilled water). For each density evaluated, the larvae were transferred using a Pasteur pipette without causing injury. We assessed the number of larvae consumed (i.e., larva carcasses floating on the water surface or in the bottom of the container) at 40 min intervals for 6 consecutive hours, not only on the day after insecticide exposure but also over the subsequent 3 days [16,20]. The larval density was re-established at each evaluation, following the Holling functional response experiment [32]. In both experimental sets, 10 *Bu. tarsalis* adults (replicates) were used for each combination of insecticide concentration and prey density. All predation bioassays were performed at controlled temperature (25 ± 2 °C), humidity (60 ± 2%), and photoperiod (12 h of light phase) conditions.

### 2.4. Statistical Analysis

For the data obtained in the survival bioassays, we applied Kaplan–Meier estimators (Log-Rank method) using the SigmaPlot 12.5 software (Systat Software, San Jose, CA, USA). Predation data were submitted to analysis of variance with repeated measures to determine the effects of insecticide, larval density, and recovery time using the SAS software (SAS Institute, Cary, NC, USA, 2010). The number of preyed larvae in each 40 min interval on the first day after exposure and the daily number of consumed *Ae. aegypti* larvae were used as replicates (within sample variation) to avoid problems of temporal pseudo-replication [33]. The general linear model (GLM) procedure with the PROFILE statement was used for this analysis using SAS software (SAS Institute 2010). Additionally, regression analyses were performed to obtain the equations of larval consumption curves over time using the curve-fitting procedure in SigmaPlot 12.5 software. We chose the regression model based on parsimony, lower standard errors, and steep increases in R2 with model complexity. We assessed the assumptions of normality and homogeneity of variance using the UNIVARIATE procedure (SAS Institute 2010), and no data transformations were necessary. Total consumption averages at the end of 4 days were compared using the Mann–Whitney Rank Sum Test (*p* < 0.05) using the SigmaPlot 12.5 software.

## 3. Results

### 3.1. Toxicity of Diflubenzuron to Three Naturally Occurring Mosquito Larvae Predators

**Figure 1:**
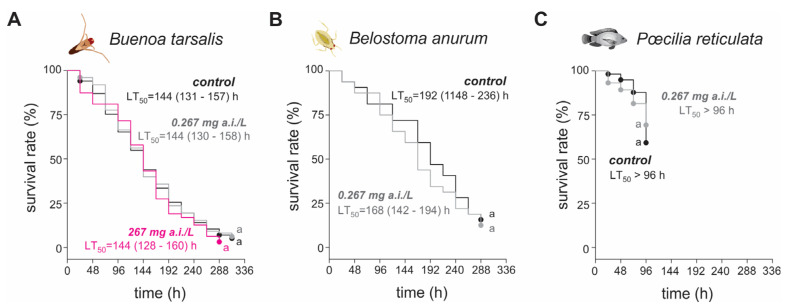
Survival rate of *Buenoa tarsalis* adults (**A**), *Belostoma anurum* second instar nymphs (**B**), and *Poecilia reticulata* adults (**C**) when subjected to diflubenzuron exposure. Treatments with the same letter do not differ, according to HolmSidak’s test (*p* > 0.05). LT_50_ = median lethal time (hours).

Survival analysis showed no differences between diflubenzuron concentrations and control for *Bu. tarsalis* adults (Log-Rank: *χ*^2^ = 0.8, *df* = 2, *p* = 0.7) (Figure 1A), even at a concentration as high as 1000-fold the recommended concentration for mosquito control, i.e., 267 mg/L. Interestingly, *Bu. tarsalis* showed a similar median lethal time (LT_50_) of 144 h for all diflubenzuron concentrations (i.e., 0.267 and 267 mg/L) and controls (Figure 1A). The LT_50_ value for *Be. anurum* exposed to diflubenzuron at 0.267 mg/L was 168 h (Figure 1B), and no difference was observed between control and diflubenzuron treatment (Log-Rank: *χ*^2^ = 0.5, *df* = 1, *p* = 0.5). Similarly, there is no statistically significant difference between the estimated median lethal time for diflubenzuron-unexposed and diflubenzuron-exposed (0.267 mg/L) *Po. reticulata* adults (Log-Rank: *χ*^2^ = 0.2, *df* = 1, *p* = 0.7) (Figure 1C). At 96 h, the mortality of *Po. reticulata* was less than 50%, both for the control and diflubenzuron (Figure 1).

### 3.2. Effect of Diflubenzuron on the Predatory Abilities of Bu. tarsalis Adults

**Figure 2:**
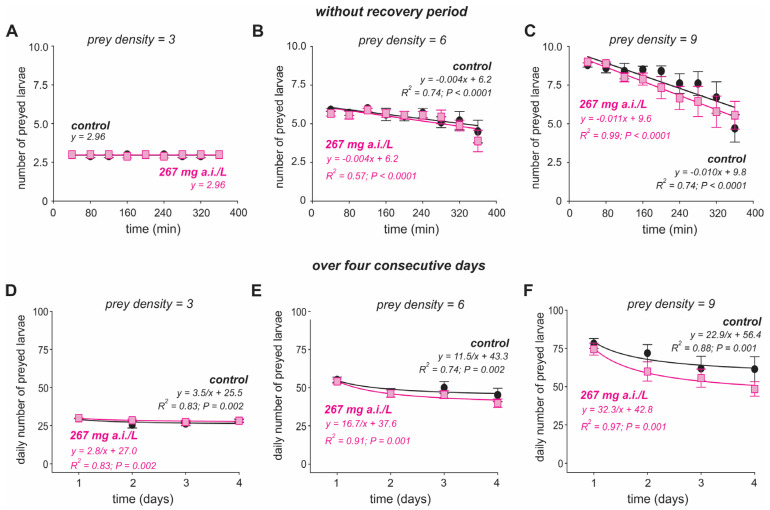
Predation of second instar (L2) larvae of *Aedes aegypti* by the backswimmer *Buenoa tarsalis* after exposure to diflubenzuron for 24 h. Number of *Ae. aegypti* larvae consumed were recorded immediately after insecticide exposure, i.e., without recovery period (**A**–**C**), and over the three more days (**D**–**F**). Three larval densities were used: three (a panel), six (b panel), and nine (c panels) larvae/100 mL. Symbols show the average number (±standard error, SE) of larvae preyed upon by each backswimmer.

For the exposure time of 24 h, our analysis of variance with repeated measures revealed that the insecticide (*F*_(1,2)_ = 0.69, *p* = 0.41) and its interactions with larval density (*F*_(1,4)_ = 0.38, *p* = 0.69), with time (*F*_(16,220)_ = 0.61, *p* = 0. 76), and with larval density and time (*F*_(32,407)_ = 0.63, *p* = 0.85) did not affect the number of larvae consumed by the *Bu. tarsalis* (Table 1, Figure 2A–C). However, the number of larvae consumed by *Bu. tarsalis* was affected by the larval density (*F*_(1,2)_ = 157.9, *p* < 0.0001), by time (*F*_(8,110)_ = 6.95, *p* < 0.0001), and by the interaction between time and larval density (*F*_(16,220)_ = 3.88, *p* < 0.0001) (Table 1, Figure 2A–C). When faced with the prey density of three larvae, *Bu. tarsalis* consumed approximately three larvae over time (Figure 2A). The number of larvae consumed by *Bu. tarsalis* reduced in a similar manner for diflubenzuron-unexposed and -exposed individuals in densities of both six (Figure 2B) and nine larvae/100 mL (Figure 2C).

The daily number of larvae consumed over four days for 24 h diflubenzuron-exposed *Bu. tarsalis* was affected by larval density (*F*_(1,2)_ = 63.7, *p* < 0.0001), time (*F*_(3,115)_ = 15.5, *p* < 0.0001), and the interaction between time and larval density (*F*_(6,230)_ = 4.37, *p* = 0.0006) (Table 2, Figure 2D–F). However, insecticide (*F*_(1,2)_ = 1.78, *p* = 0.19), the interactions of larval density with insecticide (*F*(1,4) = 1.19, *p* = 0.31), insecticide with time (*F*_(6,230)_ = 0.94, *p* = 0.43), and insecticide with larval density and time (*F*_(12,305)_ = 0.75, *p* = 0.61) were not significant (Table 2, Figure 2D–F). The daily number of consumed larvae by *Bu. tarsalis* was reduced in a similar manner over time for all treatments and larvae densities (Figure 2D–F). At the end of four days, there was no significant difference between the total number of larvae preyed on by diflubenzuron-unexposed and -exposed *Bu. tarsalis* for all larvae densities (Supplementary Figure S1).

**Figure 3:**
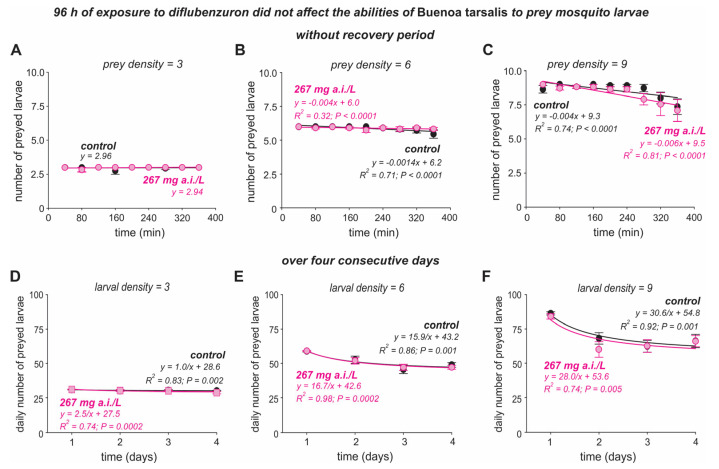
Predation of *Aedes aegypti* second instar (L2) larvae by backswimmer *Buenoa tarsalis* after exposure to diflubenzuron for 96 h. Number of *Ae. aegypti* larvae preyed upon were recorded immediately after insecticide exposure, i.e., without recovery period (**A**–**C**), and over the three more days (**D**–**F**). Three larval densities were used: three (a panels), six (b panels), and nine (c panels) larvae/100 mL. Symbols show the average number (±standard error, SE) of larvae preyed upon by each backswimmer.

Regarding the 96 h exposure period, no significant differences in the number of larvae consumed by *Bu. tarsalis* were recorded for the insecticide (*F*_(1,2)_ = 0.62, *p* = 0.43) and its interactions with larval density (*F*_(1,4)_ = 0.99, *p* = 0.38), with time (*F*_(16,220)_ = 1.57, *p* = 0.16), and with time and larval density (*F*_(32,407)_ = 0.91, *p* = 0.55) (Table 3, Figure 3A–C). However, the larval density (*F*_(1,2)_ = 1355.9, *p* < 0.0001) and time (*F*_(8,110)_ = 2.71, *p* = 0.014) affected the number of larvae consumed by *Bu. tarsalis* (Table 3, Figure 3A–C). The daily number of mosquito larvae consumed by *Bu. tarsalis* over four days after being diflubenzuron-exposed for by 96 h was affected by larval density (*F*_(1,2)_ = 211.3, *p* < 0.0001), time (*F*_(3,115)_ = 37.4, *p* < 0.0001), and the interaction between time and larval density (*F*_(6,230)_ = 10.1, *p* < 0.0001) (Table 4, Figure 3D–F). However, the insecticide (*F*_(1,2)_ = 0.41, *p* = 0.52) and its interactions with larval density (*F*_(1,4)_ = 0.22, *p* = 0.80), with time (*F*_(6,230)_ = 1.05, *p* = 0.38), and with larval density and time (*F*_(12,305)_ = 0.70, *p* = 0.65) were not significant (Table 4, Figure 3D–F).

As shown in the 24 h exposure period, the abilities of *Bu. tarsalis* exposed to diflubenzuron for 96 h to prey on mosquito larvae were not affected just after the exposure period (Figure 3A–C) or over four consecutive days (Figure 3D–F). At the end of four days, there were no significant differences between the total number of larvae preyed on by *Bu. tarsalis* untreated and treated with diflubenzuron for all larvae densities (Supplementary Figure S1).

## 4. Discussion

Here, we evaluated the impact of the insecticide diflubenzuron on the survival and predatory abilities of non-target aquatic organisms. Diflubenzuron at the concentration recommended for mosquito larvae control (0.267 mg/L) did not affect the survival of three naturally occurring aquatic predators, backswimmers *Bu. tarsalis*, water bugs *Be. Anurum*, and fish *Po. reticulata*. Furthermore, we showed that even at a high concentration (267 mg/L), diflubenzuron did not impact the survival of the backswimmer *Bu. tarsalis*. We also demonstrated that sublethal exposures to diflubenzuron present no harmful effects on the abilities of *Bu. tarsalis* to prey upon *Ae. aegypti* larvae.

Our findings indicate that the applications of diflubenzuron may not impact the ecological services, i.e., predation of mosquito larvae, provided by *Bu. tarsalis*. Nevertheless, it is worth noting that other studies with biocontrol predators reported diflubenzuron sublethal effects, as in the case of the odonatan *Ischnura elegans*, which had egg hatching and larval growing negatively impaired [34]. It is worth noting, however, that our study used *Ae. aegypti* larvae focusing on prey–predator–insecticide interactions under worst-case conditions (e.g., overestimated recommendations for field–rice paddies applications, urban runoff) to test our ecological risk hypothesis, and this model may not necessarily represent a realistic scenario. However, mosquito larvae (including other *Aedes* species) likely encounter their predator in artificial and natural water reservoirs [11,18,35,36,37,38].

The survival analysis of *Bu. tarsalis* revealed that even at a concentration 1000-fold higher than the field dose, diflubenzuron proved to be safe; this is the first report on the impact of this insecticide on *Bu. tarsalis*. The exposure (48 h) of the backswimmer *Anisops sardeus* to diflubenzuron demonstrated a LC_50_ of approximately 2 mg/L, classifying the insecticide as moderately toxic for this species [6]. In contrast, previous investigations [8] estimated an LC_50_ of 2.77 mg/L and LC_90_ of 860 mg/L of diflubenzuron for species of the *Buenoa* genus in a 96 h exposure. This wide range of actions suggests that sensitivity to insecticide may vary between different species of the *Buenoa* genus, and, according to our results, *Bu. tarsalis* is a species with high tolerance to diflubenzuron exposures. It is noteworthy that we exposed adult backswimmers to diflubenzuron, and, at this development stage, insects may lack target receptors (e.g., chitin synthase), which consequently contribute to low sensitivity.

There are still no studies regarding the impact of diflubenzuron on the giant water bug *Be. anurum*. Belostomatids have a wide range of prey, including the larval stages of disease-carrying mosquitoes, such as the genera *Aedes* and *Culex* [11,18,19,20]. Although exposure to field doses of diflubenzuron has been sublethal to *Be. anurum* nymphs, studies indicate that this species is very susceptible to exposure to insecticides. The pyrethroid insecticide deltamethrin was toxic to *Be. anurum* nymphs exhibiting a LC_50_ of only 90.9 µg a.i./L [21]. Exposure to deltamethrin is expected to have a more immediate impact on insect occurrence, as the insecticide is neurotoxic [39], whereas growth regulators cause death during the ecdysis process [40]. Exposure to the juvenile hormone analogue pyriproxyfen, which, like diflubenzuron, belongs to the class of growth regulators, proved to be more toxic, reducing the mean insect survival time even at concentrations as low as 2.5 µg a.i./L [22]. Thus, our results indicate that the diflubenzuron has greater selectivity in its mode of action, having less impact on non-target organisms. However, more studies should be carried out investigating if diflubenzuron can impact *Be. anurum* predatory capacity, as was done with *Bu. tarsalis.*

The high selectivity factor of diflubenzuron can help to explain the fact that *Po. reticulata* fish did not suffer any reduction in their survival abilities when exposed to diflubenzuron’s recommended field dose. Diflubenzuron has been classified as non-toxic to *Po. reticulata*, exhibiting an LC_50_ of 151.98 mg a.i./L [27]. However, different fish species may show different susceptibility to the insecticide. Exposure of the species *Oreochromis niloticus* and *Hyphessobrycon eques* resulted in much lower LC values, in the range of 10 mg a.i./L after 48 h of exposure [7], whereas exposure of *Piaractus mesopotamicus* to 70 mg a.i./L did not cause mortality in a study of the sublethal effects of diflubenzuron [41]. The fact that the survival of *Po. reticulata* was not impacted by the field dose of diflubenzuron is a promising result, as this tropical species has been introduced as a biological control agent for mosquitoes in aquatic environments [24,25]. Future studies should be done evaluating if diflubenzuron has any sublethal effects in *Po. reticulata* that could impair its ecological function.

Most studies on the impact of diflubenzuron on aquatic and terrestrial organisms are focused on its lethal effects as well as its impact on the taxonomic diversity of species present in contaminated environments [8,34,42,43]. However, recent works have reported the occurrence of sublethal effects in response to treatments with diflubenzuron, including reduction in food consumption and the development of bee microcolonies [44], behavioral and morphological changes in *Ae. aegypti* larvae [45], and reduction in egg hatching and fecundity in emerged adults of *Eriopis connexa* [46]. Here, although a diflubenzuron concentration equivalent to 1000-fold the field dose recommendation did not cause any mortality in *Bu. tarsalis*, we further investigated whether two such sublethal exposures (i.e., 24 h and 96 h) would affect the abilities of these aquatic predators to consume mosquito larvae.

Our behavior assays demonstrated that *Bu. tarsalis* adults are highly voracious predators of mosquito larvae, especially when facing high larval densities. Such high prey consumption by *Bu. tarsalis* is reminiscent of a previous behavior study [47]; however, the predatory abilities were examined under a reduced time regime. Although it is known that backswimmers are avid predators, we found that diflubenzuron did not affect the abilities of backswimmers to prey upon mosquito larvae independently of sublethal exposure periods and prey densities (i.e., 3, 6, or 9 larvae/100 mL distilled water). Backswimmers clearly caught more prey when exposed to high larval densities, which can be explained by the functional response (i.e., the relationship between the number of prey that a predator consumes per unit of time and the density of its prey) of this predator and other *Buenoa* species [17,32,47]. Here, since we did not conduct typical functional response curves, which are characterized by higher prey densities and extended evaluation periods, for both diflubenzuron-unexposed and diflubenzuron-sublethally-exposed *Bu. tarsalis*, we acknowledge that, while it is unlikely, we cannot completely exclude the possibility of unintended effects on the predatory abilities of *Bu. tarsalis*. In our investigations, at low prey density, the number of consumed prey did not reduce over time, indicating that a higher number of prey is required to reach a satiation level. Indeed, such a satiation level may explain the effect of time (reduced number of consumed prey over the period of evaluation) on the intermediate and high larval densities.

Backswimmers are well known for their sit-and-wait predatory behavior (i.e., ambush predators) on the water surface; they can, however, also switch their predatory strategy and actively explore the water column, using their forelegs to rapidly capture prey [48]. The unaltered foraging performance in our findings indicates that diflubenzuron did not impact the locomotion patterns of *Bu. tarsalis*. Exposure to pyrethroids, however, disrupts crucial locomotory behaviors of aquatic predators, and the outcome of this effect can translate into a reduction in predatory ability and other biological processes, such as anti-predator behavior and mating [13,21,47]. Thus, our behavioral endpoints support that diflubenzuron is safe to backswimmers because it does not alter the predatory abilities at high concentrations. Although our results suggest that diflubenzuron, a commonly utilized insecticide/acaricide, may not represent a potential risk for aquatic predators, further studies are still required to evaluate whether sublethal exposure would affect prey–predator interactions using other non-target predators and mosquito larvae. For instance, other notonectids (e.g., *Corixa punctata*, *Anisops sardea,* and *Notonecta glauca*) have shown to be much more susceptible to diflubenzuron, as exposure (<96 h) to concentrations as low as 16 g/L, which is approximately 16-fold lower than the field recommendation for controlling mosquito larvae in Brazil, resulted in approximately 15% mortality [6,49]. Potential unintended sublethal effects of these organisms that survived exposures to such low concentrations would contribute to refine our understanding of the diflubenzuron effects on the ecological services provided by those beneficial non-target aquatic organisms.

## 5. Conclusions

Our findings indicate that diflubenzuron is not toxic to *Bu. tarsalis* adults, *Be. anurum* nymphs, and *Po. reticulata* adults at concentrations as high as 0.267 mg a.i./L., which corresponds to the dose recommendation for field applications. Furthermore, we observed no adverse effects on the predatory performance of *Bu. tarsalis* when it was sublethally exposed to diflubenzuron (i.e., 267 mg a.i./L., the equivalent to 1000-fold the field dose recommendation) independently of exposure periods and prey densities. Altogether, diflubenzuron can represent a safe tool to be employed into mosquito control programs in freshwater ecosystems, especially those where backswimmers coexist with mosquito larvae.

## Figures and Tables

**Table 1:** Analysis of variance with repeated measures over time for the mean number of *Aedes aegypti* second instar larvae (L2) preyed upon (at 40 min intervals) by *Buenoa tarsalis* adults after 24 h exposure to diflubenzuron (267 mg a.i./L).

**Sources of Variation**				
	*df*	*F*	*p*	
**Between Samples**				
Insecticide (I)	1	0.69	0.41	
Density (D)	2	157.9	<0.0001 ^1^	
I × D	2	0.38	0.69	
Error	51	-	-	
	*df_den_/df_num_*	*Wilks’ lambda*	*F*	*p*
**Within Samples**				
Time (T)	44/8	0.44	6.95	<0.0001 ^1^
T × I	44/8	0.90	0.61	0.76
T × D	88/16	0.34	3.88	<0.0001 ^1^
T × I × D	88/16	0.80	0.63	0.85

^1^ Significant at *p* < 0.05.

**Table 2:** Analysis of variance with repeated measures over time for the predation abilities (daily number of larvae consumed over four consecutive days) of *Buenoa tarsalis* adults after 24 h exposure to diflubenzuron (267 mg a.i./L).

**Sources of Variation**				
	*df*	*F*	*p*	
**Between Samples**				
Insecticide (I)	1	1.78	0.19	
Density (D)	2	63.7	<0.0001 ^1^	
I × D	2	1.19	0.31	
Error	51	-	-	
	*df_den_/df_num_*	*Wilks’ lambda*	*F*	*p*
**Within Samples**				
Time (T)	49/3	0.52	15.5	<0.0001 ^1^
T × I	49/3	0.95	0.94	0.43
T × D	98/6	0.62	4.37	0.0006 ^1^
T × I × D	98/6	0.91	0.75	0.61

^1^ Significant at *p* < 0.05.

**Table 3:** Analysis of variance with repeated measures over time for the mean number of Aedes aegypti second instar larvae (L2) preyed upon (at 40 min intervals) by *Buenoa tarsalis* adults after 96 h exposure to diflubenzuron (267 mg a.i./L).

**Sources of Variation**				
	*df*	*F*	*p*	
**Between Samples**				
Insecticide (I)	1	0.62	0.43	
Density (D)	2	1355.9	<0.0001 ^1^	
I × D	2	0.99	0.38	
Error	63	-	-	
	*df_den_/df_num_*	*Wilks’ lambda*	*F*	*p*
**Within Samples**				
Time (T)	56/8	0.72	2.71	0.014 ^1^
T × I	56/8	0.82	1.57	0.16
T × D	112/16	0.67	1.58	0.09
T × I × D	112/16	0.78	0.91	0.55

^1^ Significant at *p* < 0.05.

**Table 4:** Analysis of variance with repeated measures over time for the predation abilities (daily number of consumed larvae over four consecutive days) of *Buenoa tarsalis* adults after 96 h exposure to diflubenzuron (267 mg a.i./L).

**Sources of Variation**				
	*df*	*F*	*p*	
**Between Samples**				
Insecticide (I)	1	0.41	0.52	
Density (D)	2	211.3	<0.0001 ^1^	
I × D	2	0.22	0.80	
Error	63	-	-	
	*df_den_/df_num_*	*Wilks’ lambda*	*F*	*p*
**Within Samples**				
Time (T)	61/3	0.35	37.4	<0.0001 ^1^
T × I	61/3	0.95	1.05	0.38
T × D	122/6	0.45	10.1	<0.0001 ^1^
T × I × D	122/6	0.93	0.70	0.65

^1^ Significant at *p* < 0.05.

## Data Availability

The original contributions presented in this study are included in the article/Supplementary Material. Further inquiries can be directed to the corresponding authors.

## Supplementary Materials

The following supporting information can be downloaded at https://www.mdpi.com/article/10.3390/insects16040435/s1: Figure S1: Total number of *Aedes aegypti* second instar (L2) larvae preyed by backswimmers *Buenoa tarsalis* at the end of the experiment (4 days). Backswimmers adults were exposed to 267 mg/L of diflubenzuron (magenta bar) or control (black bars) for 24 (left panel) and 96 h (right panel), subsequently their ability to prey on L2 larvae was examined for four consecutive days. Backswimmers abilities were assessed at larval densities of three (low), six (intermediate) and nine (high) larvae/100 mL of water. Bars represent the average number (±standard error, SE). Means grouped under the same horizontal line are not significantly different by Mann-Whitney Rank Sum test (*p* < 0.05).
