# Supplementary material for: Diflubenzuron Did Not Affect the Abilities of the Backswimmer Buenoa tarsalis to Survive and Prey Upon Larvae of Aedes aegypti
Source: Insects. 2025 Apr 21;16(4):435. doi: 10.3390/insects16040435 (PMC12028109; doi:10.3390/insects16040435)
Supplement: Supplementary file 1 [file insects-16-00435-s001.zip › insects-3533050-supplementary.pdf]

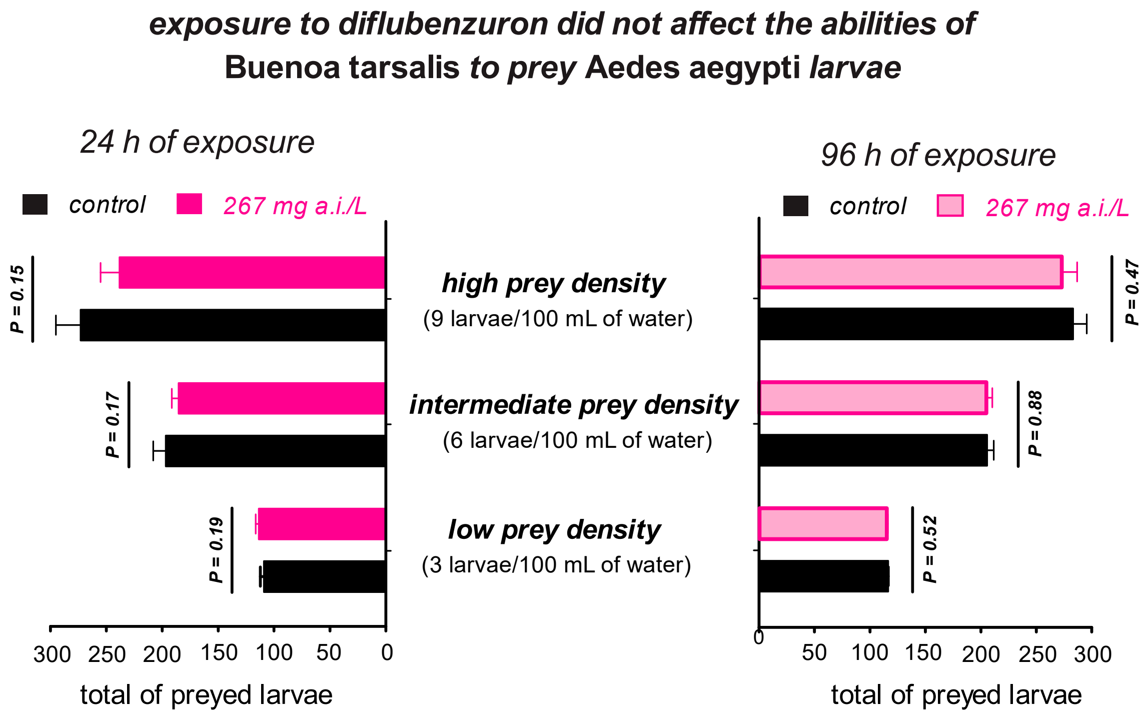

Figure S1: Total number of *Aedes aegypti* second instar (L2) larvae preyed by backswimmers *Buenoa tarsalis* at the end of the experiment (4 days). Backswimmers adults were exposed to 267 mg/L of diflubenzuron (magenta bar) or control (black bars) for 24 (left panel) and 96 h (right panel), subsequently their ability to prey on L2 larvae was examined for four consecutive days. Backswimmers abilities were assessed at larval densities of three (low), six (intermediate) and nine (high) larvae/100 mL of water. Bars represent the average number (± standard error, SE). Means grouped under the same horizontal line are not significantly different by Mann-Whitney Rank Sum test ($p < 0.05$).
